# Confirmation by necropsy of a high prevalence of porcine cysticercosis in a rural district of Madagascar

**DOI:** 10.1017/S0031182023000653

**Published:** 2023-08

**Authors:** Diana Edithe Andria Mananjara, Mihajamanana Rakotoarinoro, Valisoa C. Rakotoarison, Modestine Raliniaina, Nivohanitra P. Razafindraibe, Claudia Ravonirina, Tantely Randriamparany, Harentsoaniaina Rasamoelina-Andriamanivo, Raphaël Rakotozandrindrainy, Eric Cardinale, Marshall W. Lightowlers, Meritxell Donadeu, Kabemba E. Mwape

**Affiliations:** 1 National Center for Applied Research on Rural Development (FOFIFA), BP04 Rue Farafaty Ampandrianomby, Antsirabe, Antananarivo 101, Madagascar; 2 Veterinary Services, Ministry of Agriculture and Livestock, Madagascar; 3 Regional Directorate of Agriculture and Livestock Vakinankaratra, Madagascar; 4 National Veterinary Diagnostic Laboratory, Anosimasina Itaosy, Antananarivo 102, Madagascar; 5 Indian Ocean Commission/SEGA-One Health Network, Ébène, Mauritius, Madagascar; 6 University of Antananarivo, Antananarivo 101, Madagascar; 7 CIRAD, Montpellier, France; 8 University of La Réunion; 9 Department of Biosciences, Melbourne Veterinary School, 250 Princes Highway, Werribee, Victoria 3030, Australia; 10 Initiative for Neglected Animal Diseases (INAND), Constantia Park, Pretoria, South Africa; 11 Department of Clinical Studies, School of Veterinary Medicine, University of Zambia, Lusaka 10101, Zambia

**Keywords:** cysticercosis, Madagascar, necropsy, pig, prevalence, *Taenia solium*

## Abstract

Neurocysticercosis is recognized as an important health issue in the Malagasy population. To date, investigations into prevalence of infection with the causative agent, *Taenia solium*, in the parasite's natural animal intermediate hosts, have relied on serological methods which have been found to be non-specific. We determined the prevalence of porcine cysticercosis among pigs from a contiguous area of the Betafo and Mandoto administrative districts, Vakinankaratra Region, Madagascar. One hundred and four slaughter-weight pigs were examined by detailed necropsy examination including slicing of the heart, tongue, masseter muscles, diaphragm and carcase musculature. Thirty-seven animals (35.6%) were found infected with *T. solium*, representing one of the highest rates of infection ever reported, worldwide. These findings highlight the importance of *T. solium* in Madagascar and support the need for increased efforts to prevent the parasite's transmission to reduce its burden on the health of the Malagasy population.

## Graphical abstract

**Figure:**
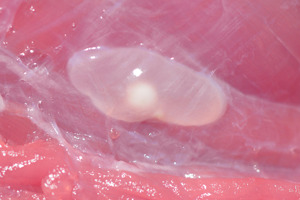

## Introduction

Cysticercosis in humans caused by infection with the larval stage of the parasite *Taenia solium* is one of the number of diseases recognized by the World Health Organization as neglected tropical diseases (World Health Organization, 2015). Infections in the brain cause neurocysticercosis, which in endemic areas is considered to be the most frequent preventable cause of seizure disorders (Ndimubanzi *et al*., 2010). The parasite is transmitted by pigs, in which it is ranked as the most important foodborne parasitic infection from a global perspective (Robertson *et al*., 2013; Havelaar *et al*., 2015).

*Taenia solium* is endemic in Madagascar, where it has been reported since 1901 (Migliani *et al*., 2000; Carod and Dorny, 2020). Neurocysticercosis is widespread in Madagascar (Andriantsimahavandy *et al*., 2003), with infection levels in school-aged children a particular concern (Zafindraibe *et al*., 2017; Carod *et al*., 2021). It is the main cause of secondary childhood epilepsy (Carod and Dorny, 2020) and a major cause of late-onset epilepsy (Andriantsimahavandy *et al*., 1997) in Madagascar.

The prevalence of porcine cysticercosis in Madagascar has been the subject of numerous studies, where infection has been determined either by meat inspection procedures or serology (Rasamoelina-Andriamanivo *et al*., 2014; Porphyre *et al*., 2015, 2016). Meat inspection procedures are understood to underestimate the prevalence of porcine cysticercosis because of a bias towards identification of heavily infected carcases only (Boa *et al*., 2002; Dorny *et al*., 2004; Phiri *et al*., 2006; Sithole *et al*., 2019). Initial descriptions of serological assays for porcine cysticercosis, such as the detection of antibodies detected in enzyme-linked immunoelectrotransfer blot (Gonzalez *et al*., 1990; Tsang *et al*., 1991) or circulating parasite-derived antigens (Dorny *et al*., 2004) suggested that both methods provided both highly specific and sensitive diagnosis. More recently, however, both methods have been evaluated critically against diagnosis at necropsy, the gold standard, and both were found to greatly overestimate infection levels due to poor specificity (Gavidia *et al*., 2013; Chembensofu *et al*., 2017; Sithole *et al*., 2019; Kabululu *et al*., 2020). These deficiencies limit the reliability of currently available data on the prevalence of porcine cysticercosis in Madagascar. To our knowledge, there has not been an accurate assessment of porcine cysticercosis in pigs from Madagascar by necropsy.

In this study, we evaluated the prevalence of porcine cysticercosis in 104 randomly selected, slaughter-age pigs from the districts of Mandoto and Betafo in Vakinankaratra Region, Madagascar by detailed necropsy.

## Materials and methods

### Study design

**Figure 1.**
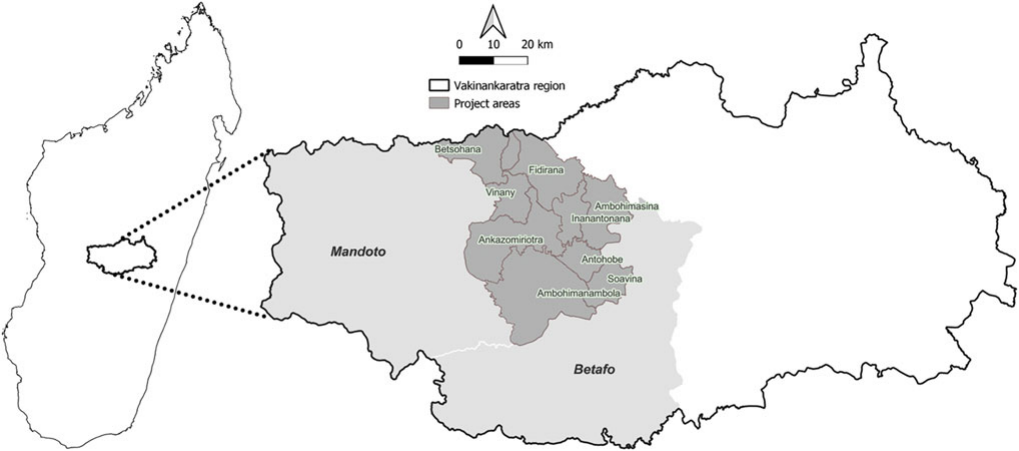
Map showing the location of the project area in central Madagascar.

The study was undertaken in a single contiguous area of central Madagascar comprising parts of the 2 administrative districts of Vakinankaratra Region, Betafo and Mandoto (Fig. 1). The area is located in the highlands of Madagascar, where 93% of the population are farmers and live in rural area (INSTAT, 2020). The site selection was based on an observed predominance of pig farming, especially in extensive system where pigs are free roaming (CREAM, 2013). In addition, local veterinarian report records indicated a high prevalence of porcine cysticercosis at meat inspection (Dr Claudia Ravonirina, pers. comm.).

A census was conducted in June 2021 to identify the characteristics of pig farming system, the number of pigs and farmers in the area, especially those who have pigs reaching slaughter weight for local consumption. Thus, a list of farmers who practice free roaming system and who have 1 or more pigs around 50 kg (average slaughter weight) per Fokontany (lowest administrative level; group of 4 or 5 villages) was obtained and used as a sampling frame for the study. Pigs for necropsy assessment were sampled at random and in proportion to the approximate number of pigs resident in each of the 48 Fokontany in which the project was undertaken. Sample size (104 animals) was calculated from a population of 45 000 pigs distributed in 20 Fokontany, each ranging from 350 animals upwards per Fokontany, with the samples assumed to be aggregated. The expected initial necropsy prevalence was assumed to be 15%. Sample size was calculated with a power of 90 and 99% confidence interval. Calculations were undertaken in R *via* Monte-Carlo simulations. One animal only was obtained from each farmer, although inadvertently 3 farmers were found to have provided 2 pigs each.

### Post-mortem procedures

The pigs were transported to Antananarivo, at the National Laboratory for Veterinary Diagnostics where they were euthanized by professional slaughter staff according to normal commercial practices. Necropsy procedures were similar to those described by Sah *et al*. (2017) and Nsadha *et al*. (2021), with the exception that the abdominal, thoracic organs (except for the heart) and the brain were not assessed for infection. This was based on recent analyses and data which indicate that *T. solium* cysticerci are rarely found in sites other than striated muscles and brain, except possibly in the case of exceptionally heavy infections (Gauci *et al*., 2019), and that animals that had undergone whole-body necropsy and had cysts in the brain also had cysts present in the muscle tissue (Lightowlers, 2020). Prior to slaughter, the weight of the animals was estimated by an experienced animal handler. Following slaughter, the abdominal organs were discarded, except for the heart. All carcase parts were carefully labelled to ensure the identity of the individual animal was retained. The masticatory muscles (internal and external, both left- and right-hand side), diaphragm and tongue were dissected from the head and the carcase bisected longitudinally. The carcase parts were refrigerated overnight at 4°C.

### Examination for *T. solium* cysts

The muscles of the right-hand side of the carcase as well as the masseters, tongue, full diaphragm and heart were dissected from the bodies and sliced by hand at approximately 3 mm intervals to reveal all cysts. Where the number of cysts in the carcase musculature was determined to be >1000, the number was not counted and simply recorded as >1000. Where no cysts were identified, the left-hand side of the carcase was assessed for possible infection in a similar way. The total number of cysts in the animals was estimated by inclusion of a figure for the left-hand side carcase musculature being a doubling of the number found in the right-hand side musculature. Cysticerci were recorded as viable where they were translucent vesicles filled with transparent fluid and having a visible white scolex. Non-viable lesions were recorded separately in cases where vesicles were non-translucent, containing a dense white or yellowish fluid and having no scolex and in cases of fibrosed or calcified lesions.

### Case definition of confirmed porcine cysticercosis

An animal was determined to be a confirmed case of porcine cysticercosis if 1 or more viable cysticerci were found in the muscles, or if more than 1 non-viable lesion was detected in the muscles.

### Data analysis

All statistical analysis was performed in R software. The prevalence of infected animal with cysts and viable cysts were calculated with their 95% confidence interval (CI). Univariate analysis through parametric *χ*^2^ test was used to compare the proportion of animal with different intensities of infection according to the viability of cysts and the sex of pigs. The significant difference threshold of 0.05 was considered.

## Results

*Taenia solium* infections detected in pigs from the study areas are detailed in Table 1. Among 104 animals that were necropsied, 37 pigs [35.6% (CI 26.4–45.6%)] were found to be infected. Among infected animals, 86% were found to harbour viable cysts (representing 30.8% of all animals necropsied). Nineteen animals (51% of the infected animals) were found to harbour both viable and non-viable cysts, with a further 5 animals (14%) having only non-viable cysts. Five animals were detected with a single, unidentified non-viable lesion in the striated muscle tissue; these individuals were not classified as being infected. Of the 2 animals inadvertently obtained from each of 3 farmers, 1 pair was uninfected, 1 had both animals infected and 1 pair had 1 infected and 1 uninfected animal.

**Table 1.** Numbers and proportions of pigs with *Taenia solium* cysts (viable or non-viable) and burden of infection detected at necropsy among animals derived from adjoining areas of Mandoto and Betafo districts, Madagascar

		Infected with viable cysts		Infected with cysts (viable or non-viable)	
Description	Number of animals necropsied	Number of animals	Percentage (95% CI)	Number of animals	Percentage (95% CI)
Male	56	15	26.8 (15.8–40.3)	17	30.4 (18.8–44.1)
1–10 cysts		3		3	
11–100 cysts		4		4	
101–500 cysts		6		6	
>500 cysts		2		4	
Female	48	17	35.4 (22.2–50.5)	20	41.7 (27.6–56.8)
1–10 cysts		5		7	
11–100 cysts		4		4	
101–500 cysts		5		4	
>500 cysts		3		5	
Total	104	32	30.8 (22.1–40.5)	37	35.6 (26.4–45.6)

**Figure 2.**
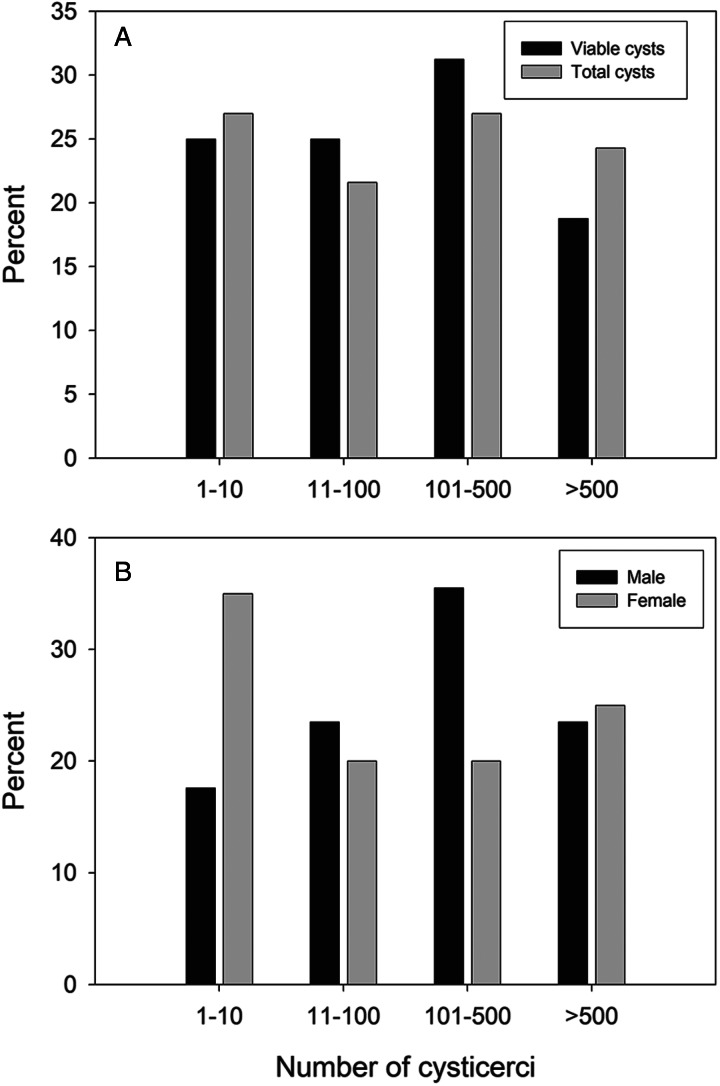
Proportions of slaughter-weight pigs from the Mandoto/Betafo districts of Madagascar infected with *Taenia solium* cysts. (A) Burden of viable and non-viable cysts; (B) burden of infection (total cyst numbers) in female and male pigs.

**Figure 3.**
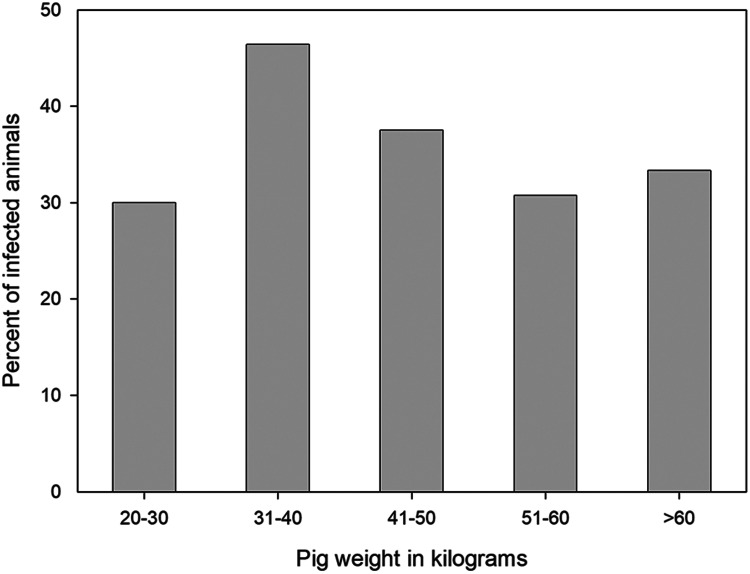
Proportion of infected pigs with *Taenia solium* from the Mandoto/Betafo districts of Madagascar in relation to their weight class at necropsy.

The proportion of animals with different intensities of infection with viable cysts, or viable plus non-viable cysts, and the proportion of total cyst numbers in male and female animals are shown in Fig. 2. There was no statistically significant difference in the proportion of animals infected with different intensities of infection with respect to either total cysts or viable cysts [*χ*^2^ test for trend (Abramson, 2004) and Fisher's exact test]. Similarly, there was no statistically significant difference in the proportion of animals of different weight classes, as shown in Fig. 3, in relation to their intensity of infection (total cysts).

Among infected pigs (37 pigs), cysts (viable and non-viable) were found in the carcase musculature (97%), tongue (70%), masseters (62%), diaphragm (54%) and heart (43%).

## Discussion

A high rate of *T. solium* infection in Malagasy pigs was found for the first time using a specific and accurate methodology. Several previous reports have described porcine cysticercosis in Madagascar using serological methods that are now considered to have poor specificity (Lightowlers *et al*., 2016). The presence of the parasite and its importance for the human population have been evident from the level of neurocysticercosis in the Malagasy population, which has been apparent for many years (Carod and Dorny, 2020).

Among the 104 slaughter-age animals that were necropsied, 37 pigs (35.6%) were found to be infected with *T. solium*. High rates of porcine cysticercosis have been detected at necropsy in pigs from other parts of the world, particularly Zambia (Dorny *et al*., 2004; Chembensofu *et al*., 2017), Mozambique (Pondja *et al*., 2012), Nepal (Sah *et al*., 2017) and Peru (Gonzalez *et al*., 1990). The rate of infection in the pigs examined in this study ranks Madagascar as among the highest rates of infection ever recorded. Risk factors for *T. solium* transmission occur in Madagascar, such as free-roaming pigs, unsupervised home slaughter, consumption of undercooked pork and open-air defecation in rural areas (Rasamoelina-Andriamanivo *et al*., 2013); however, it is unclear why the prevalence of porcine cysticercosis was particularly high in the study area compared with other regions of the world where similar risk factors pertain.

The precise burden in the infected animals that were necropsied animals in this study was not determined. After detailed assessment of the masseter muscles, tongue, diaphragm, heart and the right-hand side of the carcase by slicing and inspection of all the tissue, those animals in which at least 1 viable *T. solium* cysticercus was found had the number in the remaining half carcase musculature estimated by doubling that found in the right-side carcase. A similar calculation was made for the burden in the left side of the carcase after finding at least 2 non-viable lesions in the right side and other tissues examined. The brain was not examined because current evidence indicates that infections in the brain are exclusively, or almost exclusively, found only in animals where cysts are also present in the striated muscles (Lightowlers, 2020). Other organs were not examined for cysts because, although *T. solium* cysticerci have been found in a variety of organs other than striated muscle and nervous tissues, they occur relatively rarely and appear to occur principally in animals that have an exceptionally heavy burden of infection (Gauci *et al*., 2019). In this study, we were interested particularly in the proportion of animals that were infected with *T. solium*, rather than a precise determination of the burden in individual animals.

The presence of many cases of neurocysticercosis among the Malagasy population has been a concern for many years (Andriantsimahavandy *et al*., 1997; Carod and Dorny, 2020) and stimulated interest in the adoption of measures to reduce transmission of the parasite. With support from the World Health Organization, Ramiandrasoa *et al*. (2020) undertook annual mass drug administration (MDA) of the Malagasy population for 3 consecutive years with taeniacide in an area comprising approximately 95 000 inhabitants. Comparison of the levels of taeniasis before and 16 months after the last treatment indicated that the MDA had little impact on transmission of the parasite. Other attempts to reduce transmission of *T. solium* exclusively through the use of taeniacides have generally had limited impact (Lightowlers, 2013; de Coster *et al*., 2018). Mathematical modelling of *T. solium* control through the exclusive application of taeniacides annually in the human population, even for a decade, suggests that the disease would re-establish relatively rapidly (Braae *et al*., 2019).

Effective control of *T. solium* transmission will require interventions in both the pig population as well as the human population (Lightowlers, 2013; Gabriel *et al*., 2017; Braae *et al*., 2019). Combined use of interventions in pigs and humans has led to the elimination of the parasite's transmission (Garcia *et al*., 2016; Gabriel *et al*., 2020); however, the strategies adopted to date have been complex and expensive. New, more sustainable approaches are needed for the control of *T. solium* transmission, both in Madagascar as well as in the other areas of the world where neurocysticercosis remains an unacceptable burden on the human populations.

## Data Availability

Data supporting results are provided within the article. Additional information may be available on request.
